# Bilateral increase in MEG planar gradients prior to saccade onset

**DOI:** 10.1038/s41598-023-32980-z

**Published:** 2023-04-10

**Authors:** Jasper H. Fabius, Alessio Fracasso, Michele Deodato, David Melcher, Stefan Van der Stigchel

**Affiliations:** 1 School of Psychology and Neuroscience, University of Glasgow, Glasgow, G12 8QQ UK; 2 Experimental Psychology, Helmholtz Institute, Utrecht University, 3584 CS Utrecht, The Netherlands; 3 Psychology Program, Division of Science, New York University Abu Dhabi, Abu Dhabi, United Arab Emirates

**Keywords:** Perception, Attention

## Abstract

Every time we move our eyes, the retinal locations of objects change. To distinguish the changes caused by eye movements from actual external motion of the objects, the visual system is thought to anticipate the consequences of eye movements (saccades). Single neuron recordings have indeed demonstrated changes in receptive fields before saccade onset. Although some EEG studies with human participants have also demonstrated a pre-saccadic increased potential over the hemisphere that will process a stimulus after a saccade, results have been mixed. Here, we used magnetoencephalography to investigate the timing and lateralization of visually evoked planar gradients before saccade onset. We modelled the gradients from trials with both a saccade and a stimulus as the linear combination of the gradients from two conditions with either only a saccade or only a stimulus. We reasoned that any residual gradients in the condition with both a saccade and a stimulus must be uniquely linked to visually-evoked neural activity before a saccade. We observed a widespread increase in residual planar gradients. Interestingly, this increase was bilateral, showing activity both contralateral and ipsilateral to the stimulus, i.e. over the hemisphere that would process the stimulus *after* saccade offset. This pattern of results is consistent with predictive pre-saccadic changes involving both the current and the future receptive fields involved in processing an attended object, well before the start of the eye movement. The active, sensorimotor coupling of vision and the oculomotor system may underlie the seamless subjective experience of stable and continuous perception.

## Introduction

When an object moves, its position on our retinae changes. When our eyes move, the retinal position of objects is also changed. These two possible scenarios create sensory ambiguity in the visual system: did the retinal position change because the eyes moved or because the object moved? It has been hypothesized that the visual system resolves this ambiguity by anticipating the changes in retinal input caused by eye movements^1^. Neurophysiological recordings have demonstrated that when an oculomotor command is generated by the superior colliculus, a corollary discharge (or efference copy) of the motor command is sent to the visual system which allows it to compensate for the upcoming eye movement (for review:^2–4^. In addition, neurophysiological recordings have revealed a plethora of transient changes in receptive fields before and after eye movements^5^. The most widely examined of these changes is “presaccadic remapping”, in which neurons start responding to stimuli that will fall into their receptive field after a saccade before the eyes have started to move^6^. Prior to saccade onset, receptive fields “shifts” to the ‘future receptive field’—the spatial location that will be covered by the receptive field after the saccade^7^. These findings could provide continuity between pre-and post-saccadic visual representations since the shifts ‘move’ receptive fields towards their future location. These observations have also led to theories emphasizing pre-saccadic shifts in attention^5^ or object pointers^8^, as well as suggestions of spatiotopic encoding^9^. Alternatively, given that the pattern of remapping demonstrated so far is complex and includes receptive field changes in multiple directions around saccade onset^2,10^, other theories have emphasized post-saccadic updating^11,12^ or a “soft hand-off” across the saccade^13,14^. Critical to testing these theories is the ability to characterize neural activity at a high temporal resolution around the time of saccades, which is complex to achieve for studies showing evidence for remapping in humans with, for example, fMRI^15–19^.

In this manuscript, we investigate the timing and lateralization of pre-saccadic activity evoked by visual stimuli presented in the periphery, in humans. Specifically, we aim at isolating the activity associated with the presentation of the visual stimulus or saccade execution alone, from the activity evoked by their combination.

The majority of highly time-resolved studies on neural activity in pre-saccadic visual processing have been conducted with non-human primates and single-cell recordings^2,6,10,20^. In general, they have tended to focus on the “future receptive field” which will process the object after the saccade. In humans, various EEG studies have studied pre-saccadic changes in visual responses by examining event-related potentials (ERP) ipsilateral to the presented stimulus, shortly before saccade onset^11,21–23^. The rationale for these studies is based on the idea that stimuli typically evoke contralateral responses, but when a saccade would bring the stimulus into the opposite visual hemifield, a remapped response could potentially be detected in the ERP, ipsilaterally, prior to saccade onset.

When applying this reasoning, Parks and Corballis^11,21^ compared conditions in which a visual stimulus was shown in one visual hemifield and either remained within that same hemifield after a saccade or shifted to the opposite hemifield. Their strategy was to keep the presence of a stimulus and the making of a saccade constant, and just vary whether or not the grating stimulus would switch hemifield across the saccade (in^11^, the authors also included a saccade-only condition). The authors found evidence of pre-saccadic predictive remapping in humans, compatible with previous work from neurophysiology^6,20,24^ and human fMRI studies^15,25,26^, although functional imaging prevents inferences about the exact timing of the effect, due to the slow hemodynamic response^27^. Their results reveal striking similarities between the pre-saccadic and the post-saccadic visual responses, providing evidence compatible with pre-saccadic remapping as well as post-saccadic updating and possibly, soft hand-off of information across eye movements^13,14^.

Kovalenko and Busch^22^ used a similar visual paradigm as Parks and Corballis^11^ but flashed a small probe at various perisaccadic times. These authors examined whether the timing of the probe led to different evoked responses and, similar to Parks and Corballis^11^, observed a pre-saccadic effect, although not limited to the ipsilateral channels. Finally, Peterburs and colleagues^23^ also observed a pre-saccadic effect, namely, amplitude differences for leftward and rightward saccades. However, this effect was different between leftward and rightward saccades. A positive pre-saccadic potential was only observed for leftward saccades, whereas a negative pre-saccadic potential was observed for rightward saccades. Based on these studies in humans, it becomes evident that the pre-saccadic ipsilateral response in humans is not a robust phenomenon when measured using EEG.

In order to further examine the laterality of the pre-saccadic neural activity in more detail, we made use of the high spatial and temporal resolution of magnetoencephalography (MEG) to analyze evoked neural responses to an intermediate size, stable object that was attended by participants for a change detection task. This data was previous collected in the context of examining the processing of low-level visual information across saccades^13^. In the previous multivariate analyses, we did not observe any evidence for feature-specific anticipatory processing before saccade onset. Instead, we were able to decode the pre-saccadic stimulus features until well after the saccade had ended. However, in that paper we specifically focused on multivariate differences. It is possible that the univariate effects previously been reported in EEG data are not specific to visual features, but instead reflect a more global modulation of neural activity^28–33^.

A wide variety of neural^2,6,10,15–20^ and behavioral/psychophysical^34–42^ effects have been reported during the pre-saccadic time period, prior to saccade onset. This suggests that a data-driven, whole brain analysis of the pre-saccadic time period might be important to uncover the overall pattern of neural modulation in sensory, oculomotor, working memory, attention and other brain networks. Here, we investigated these potentially predictive changes in event-related planar gradients before saccade onset. We modelled the gradients on trials in which participants were presented with a visual stimulus and made a saccade as a linear combination of two conditions where participants were either only presented a stimulus or only made a saccade (for methods using linear modeling of EEG/MEG or fMRI data and eye movements, see:^43–45^. Any residual gradient would be unique to the combination of the visual stimulus and the upcoming saccade. The benefit of using planar gradients is that their location is more easily interpretable as being directly over their dipole source^46^. To preview the results, the residual gradients were significantly larger than zero, across many sensors, starting around 112 ms prior to saccade onset. Interestingly, these predictive changes were bilateral.

## Methods

### Participants

A total of 31 right-handed participants took part in this study after giving informed consent. Two participants were excluded based on behavioral performance in the training session (N = 1) or after completing the experiment (N = 1) and one additional participant was excluded because of technical difficulties during the experiment. In total, a complete dataset of 28 participants were used for the analysis (15 male; mean age = 25.3, range = [20, 35]). This study was approved by the local ethical committee of the University of Trento. The approved methods were carried out in accordance with the Declaration of Helsinki. The same data were used in a previous publication focusing on multivariate analyses of a different temporal window of the data^13^. In this previous study, we were able to reliably decode the spatial frequency across the different conditions, revealing that the noise level is relatively low in this dataset. Furthermore, we counterbalanced the different conditions, so any potential movement related noise is evenly distributed between conditions.

### Experimental setup

The exact experimental setup is described in Fabius and colleagues (2020). In short, head coordinate frame, coil position and head shape were determined with the Fasttrack 3D digitization system (Polhemus, Colchester, VT, USA). MEG data were acquired with a Vectorview 306 channel MEG machine (Elekta Neuromag Oy, Helsinki, Finland). Eye position data were acquired with an Eyelink 1000 + at 1000 Hz, recording the left eye (SR Research Ltd., Mississauga, ON, Canada). Stimuli were projected with a PROPixx projector (VPixx Technologies, Saint-Bruno, QC, Canada) onto a translucent screen 100 cm away from the participant (refresh rate = 120 Hz; dimensions = 51 × 38 cm; resolution = 1440 × 1080 pixels). Manual responses were recorded with RESPONSEPixx (VPixx Technologies, Saint-Bruno, QC, Canada). The Eyelink extension of the Psychtoolbox^47^ was used to control the eye-tracker and control the gaze-contingent display.

### Task and stimuli

The stimuli are described in detail in Fabius and colleagues^13^. In short, stimuli were static sinusoidal gratings (radius = 4° visual angle; orientation = -30° or 30° from vertical; spatial frequency = 0.33 or 1.33 cyc/°; phase = 0 or pi, to keep luminance equal), presented at full contrast (black = 1.94 cd/m^2^, white = 142 cd/m^2^) on a uniform grey background (61.1 cd/m^2^). The center of the stimuli was located 6° below the horizontal meridian, and horizontally centered on the display.

**Figure 1:**
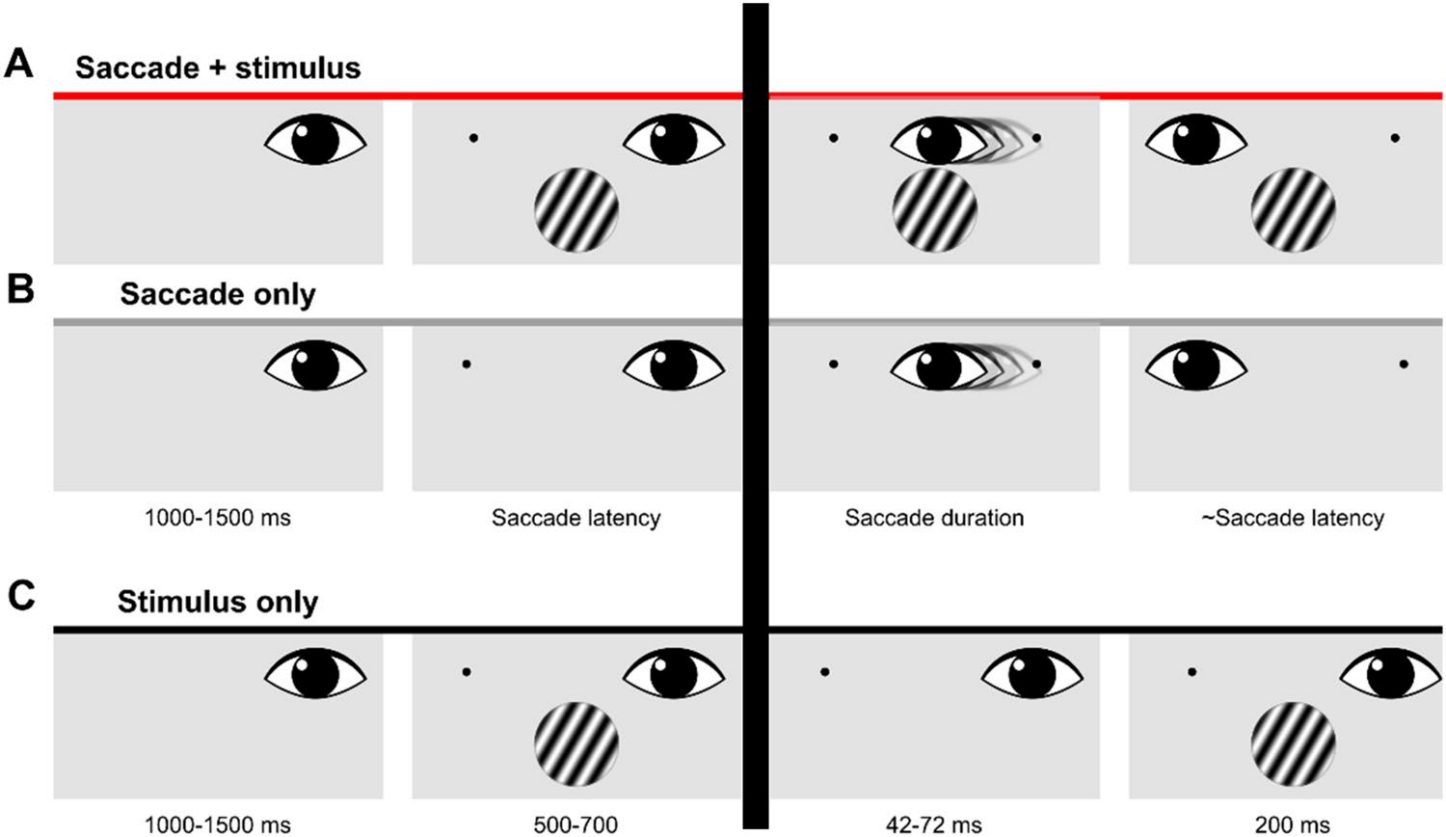
Illustration of the three conditions. Only data prior to the saccade (left of the dark vertical bar) was used for the analysis. (**A**) Timeline of events in the Saccade + stimulus condition. These trials were gaze contingent. Durations of each event are displayed at the bottom of panel B. On half of the trials, the stimulus would change its orientation during the saccade, from -30° to 30° or vice versa. Responses were given with button presses. (**B**) Timeline of events in the Saccade-only condition. In this condition participants only made a saccade; they did not perform the orientation-change detection task. (**C**) Timeline of events in the Stimulus-only condition. This condition was not gaze contingent, instead the first stimulus was briefly removed for a period that was sampled online from a distribution of saccade durations that were measured in the saccade conditions. Participants performed the same orientation-change detection task.

Participants performed trials with and without saccades (Fig. 1). These trials were presented in blocks. Within a block of saccade trials, participants fixated on the right side of the screen (7° from the midline) until a fixation target appeared 14° to the left. On two-thirds of the saccade trials (416 trials). the stimulus was also displayed after 1.0–1.5 s., simultaneously with the onset of the fixation target (Saccade + stimulus condition; Fig. 1A). On the other third of the saccade trials (208 trials), no stimulus was displayed, only the fixation target (Saccade-only condition; Fig. 1B). Participants were instructed to make a saccade to the new fixation target whether a stimulus appeared or not. No manual response was given on the Saccade-only trials. In the fixation trials, participants fixated either to the left or to the right of the fixation point (7° from the midline). For the analysis presented here, we only use the trials where participants fixated on the right side (Stimulus-only condition; Fig. 1C). After a variable interval of 1.0–1.5 s., a stimulus appeared for 0.5–0.7 s.

We opted for unilateral saccades to have more signal to play around with when averaging. Having saccades to the left and right would essentially split the dataset in half, given that our hypotheses were directional (e.g. transfer of visual information between the two hemispheres).

The analysis here focuses only on the pre-saccadic time period. In the main experiment, there were also events that occurred after the saccade was detected, which are not analyzed here. On the saccade + stimulus trials, a stimulus was presented after the saccade at the beginning of the new fixation. Using online saccade detection, the stimulus changed its orientation on half of the trials. Participants reported whether the target had changed its orientation or not with a button press, using their right hand. On the no-saccade trials, the stimulus was removed after a variable delay (matched to the saccade latencies) for 42–72 ms (normally distributed, mean = 55 ms, sd = 6 ms), and replaced with a second stimulus. The second stimulus had either the same or a different orientation. Participants reported whether the orientation had changed. These events occurring after the saccade (or, in the case of the no-saccade trials, at a time matched to the saccade latencies) were not included in the analysis.

### Preprocessing

We visually inspected all data and marked noisy channels. The native Maxwell filter of Elekta Neuromag was applied to filter signals that originated outside the MEG helmet^48–50^. Line noise (50 Hz) and its harmonics (100 and 150 Hz) were attenuated using a DFT filter on the continuous data of each run. Data were then cut into epochs from 0.5 s before until 1.5 s after S1 onset. Then, data were downsampled to 500 Hz.

The raw Eyelink recordings in the MEG datafile were converted from volt to pixels. We observed a small but consistent lag between the recordings in the MEG datafile and the Eyelink datafile of 7 ms. This lag probably originated during the digital-to-analog conversion and was compensated for by shifting all Eyelink data in the MEG datafile with 7 ms back in time with respect to the MEG data. Saccades were detected with the saccade detection algorithm of Nyström and Holmqvist^51^, with a minimum fixation duration of 40 ms and a minimum saccade duration of 10 ms.

To determine the onset of a visual event we converted the raw photodiode signal to a trinary signal—because we used three grey values: black, grey and white—by taking four linearly separated values between the minimum and maximum values of the raw signal. All values below the second boundaries were classified as black (− 1). All values between the second and third boundary were classified as grey (0). All values higher than the third boundary were classified as white (1). The absolute difference of the trinary signal was used to obtain the timing of a visual onset.

All epochs from -0.5 to 1.5 after S1 onset were visually inspected for remaining MEG artefacts (e.g. muscle activity). Epochs containing artefacts were removed (mean = 3.9%, min = 0.4%, max = 7.3%). In the conditions with saccades, epochs were included only if (1) there was a single saccade after stimulus onset and before the onset of the second stimulus, (2) the saccade endpoint was at least 4° over the vertical midline of the screen, bringing the stimulus from being entirely in the left visual field to entirely in the right visual field, and (3) the saccade endpoint was higher than 2° below the horizontal midline of the screen, keeping the stimulus entirely in the bottom visual field (mean = 8.2%, min = 0.2%, max = 28.8%). In the Fixation conditions, epochs were included only if participants (1) maintained gaze within an area of 2° visual angle around the fixation point during the entire epoch and (2) did not make microsaccades with amplitudes larger than 0.5° (mean = 4.2%, min = 0.1%, max = 21.4%).

After defining valid epochs, we restricted the inclusion in the condition even further by only including epochs when the saccade latency was between 150 and 500 ms. These latencies were selected because we intended to compare the saccade and fixation conditions. The duration of a stimulus in the fixation conditions was at least 500 ms. The lower bound of the latency inclusion was more arbitrary: we did not want saccades to be slow, but we also wanted to have epochs of a considerable length. Thus, we settled for 150 ms.

### Linear model of event-related planar gradients

We computed event-related planar gradients of the combined gradiometer data with the recordings locked to saccade onset. We used planar gradiometers because their measurements allow for a direct distinction between left and right hemisphere activity, whereas magnetometers do not. We cut epochs from − 0.6 to 0 s before saccade onset. Then, we computed the average per sensor and subsequently combined the averaged gradiometers. Lastly, we subtracted the average activity in a baseline period from − 0.6 to − 0.5 s before saccade onset. This baseline period did not include any stimulus-related activity, as we only included trials with a maximum saccade latency of 0.5 s. We did not apply any filters before or after computing the planar gradients.

**Figure 2:**
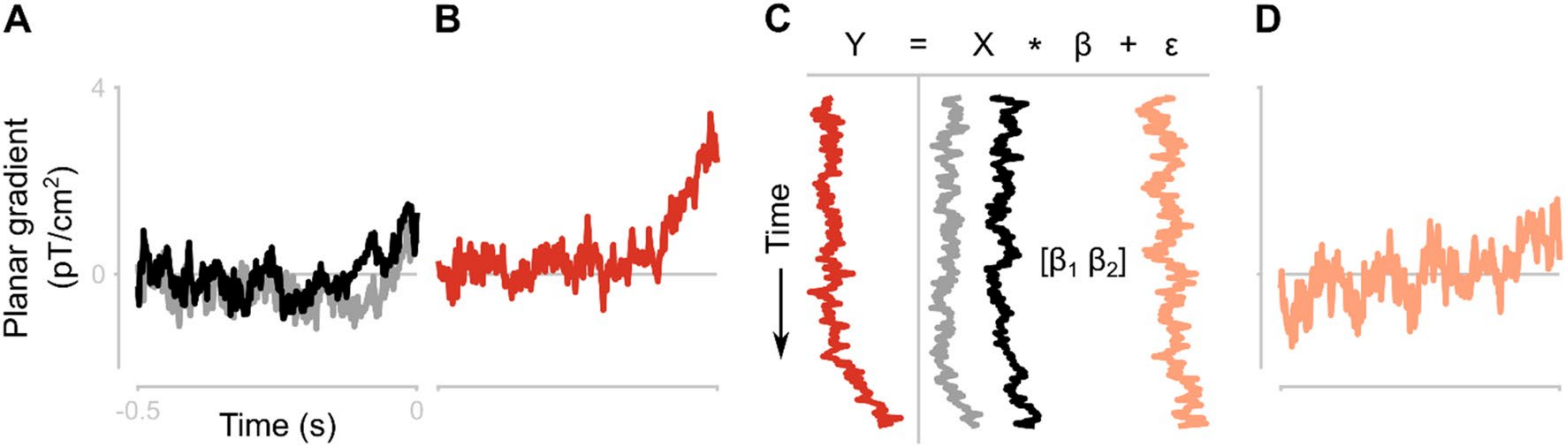
Conceptualization of the linear modelling of planar gradients in the Saccade + stimulus condition as a weighted linear combination of the Saccade-only and Stimulus-only conditions. (**A**) Example planar gradients of one combined planar gradiometer in the Saccade-only (grey) and Stimulus-only (black) conditions. Data are aligned to saccade onset. (**B**) Example planar gradient in the Saccade + stimulus condition. (**C**) Visualization of the general linear model with the planar gradients depicted in A and B. Y = Saccade + stimulus. X = [Saccade-only, Stimulus-only]. ε = residuals. (**D**) Residuals from the general linear model. The residuals represent the surplus of planar gradient in the Saccade + stimulus condition, with respect to the expected planar gradients based on the measurements in the Saccade-only and Stimulus-only conditions.

We estimated responses related to pre-saccadic remapping to be the residual planar gradients from the conditions with both a saccade and a stimulus (Saccade + stimulus) after regressing out the predicted planar gradients based on the linear, weighted combination of the saccade related planar gradients and stimulus related planar gradients (Fig. 2). For the saccade related activity, we took the saccade-locked planar gradients from the Saccade-only condition, because that condition only contains a saccade and no stimulus. For the stimulus related activity, we used the saccade-locked planar gradients from the Stimulus-only condition. We did not make a distinction between spatial frequencies of the stimulus. For each participant and each combined planar gradiometer, we constructed a linear model, estimated the parameters for the saccade related activity (β_saccade_) and the stimulus related activity (β_stimulus_). With these parameters, we estimated the predicted planar gradient (Ŷ) in the conditions with both a saccade and a stimulus (Saccade + stimulus). The residuals were the difference between the actual planar gradient and Ŷ. We then tested where the residuals deviated from zero.

There was a manual response in two out of the three conditions. We used a linear model to analyze our data, using a linear combination of ‘Stimulus-only’ and ‘Saccade-only’ conditions to account for the activity we observe in the ‘Saccade + Stimulus’ condition. It is important to note that although the manual response contribution is not present in the ‘Saccade-only’ condition, its contribution is present in the ‘Stimulus-only’ condition. Hence, when trying to account for the variability in the ‘Saccade + Stimulus’ condition, the manual response contribution is accounted for, as it is included in the ‘Stimulus-only’ condition.

### Simulating saccade onset in fixation trials

We locked epochs to the onset of the saccade (in the Saccade conditions). Because of the natural variability in saccade latencies, not all epochs had the same length. With our inclusion and exclusion criteria we assured that all trials were between 150 and 500 ms. By locking all epochs to the onset of the saccade rather than to the onset of the stimulus, stimulus-related activity would be scrambled across trials and would become convoluted when averaging over trials in one condition. On the other hand, activity related to saccade onset should become more pronounced. Trials in the Stimulus-only condition did not have a saccade. To get a comparable scrambling of stimulus evoked activity, we matched each trial in these conditions to the duration of the trials in the Saccade conditions.

We estimated kernel density distributions of saccade latencies per participant and per stimulus feature, i.e. a separate density distribution for each of the eight combinations of stimulus orientation, spatial frequency and phase. For each Stimulus-only trial, we sampled 1000 saccade onsets from these distributions, matching the stimulus features. We then computed the solutions to the linear model as described and residuals as described above. To compute statistics on the residuals, we took the median of the 1000 computed residuals per sensor and participant. To exclude the influence of any extreme value, we opted to use the median instead of the mean.

## Results

### Event-related planar gradients

**Figure 3:**
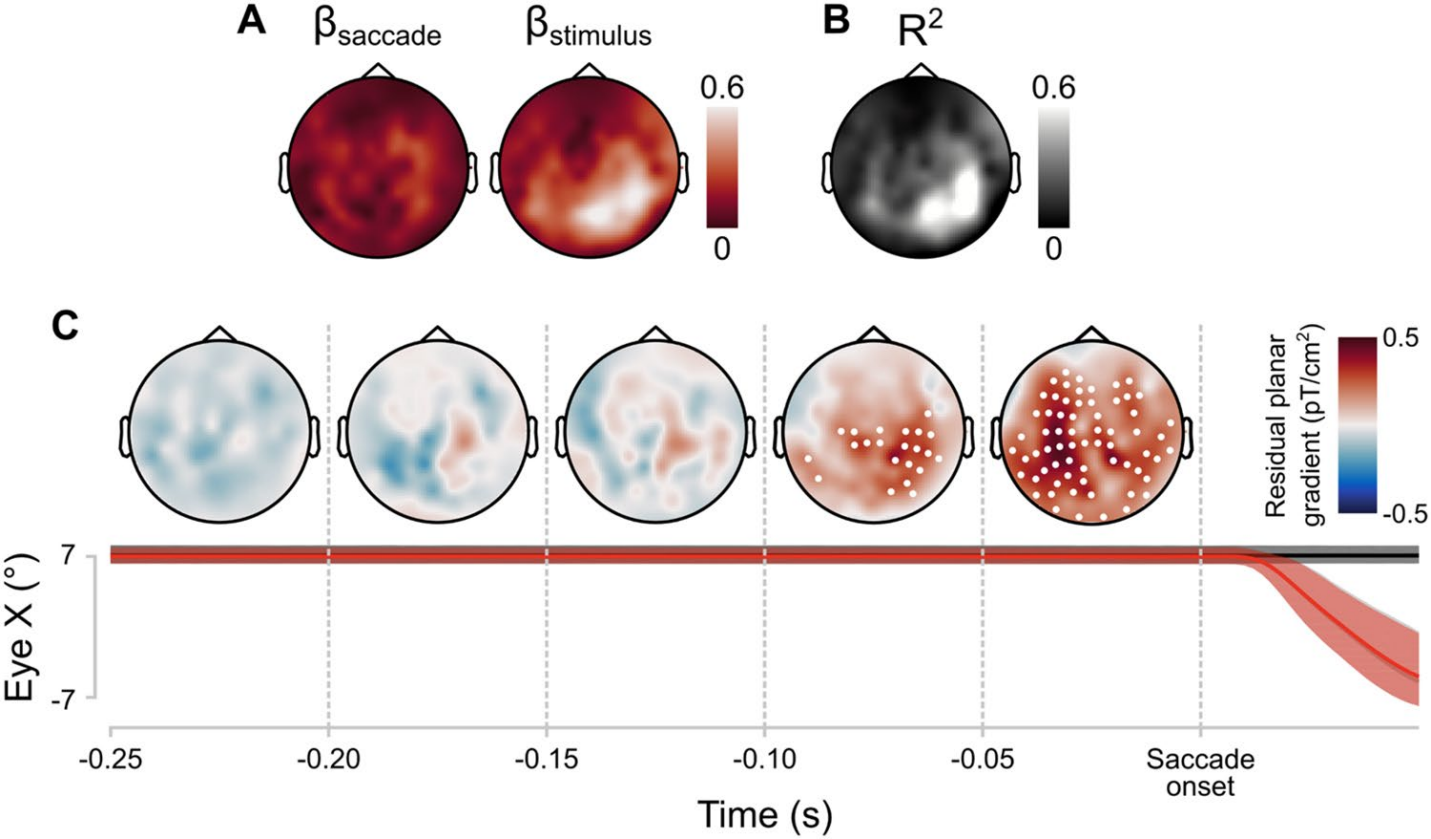
Results of linear modelling of event-related fields in the Saccade + stimulus condition as a weighted linear combination of the Saccade-only and Stimulus-only conditions. (**A**) Topographic heatmap of estimated parameters for the Saccade-only condition (β_saccade_) and the Stimulus-only (β_stimulus_). (**B**) Topographic heatmap of the proportion variance explained (R^2^) by the linear model. (**C**) Topographic heatmaps of the residual planar gradients of the linear models. Values represent group averages. Deviations from 0 were assessed with one-sample t-tests, cluster-based corrected for multiple comparisons in time and space (α = 0.05, two-tailed). Sensors with a significant deviation from 0 for at least 12.5 ms are highlighted with a white point. The average horizontal gaze position in the three conditions over time is plotted below the heatmaps. Black = Stimulus-only, grey = saccade-only, red = Saccade + stimulus (latter two overlap). Lines represent median gaze position over all trials. Shaded area is the 95% interval across all trials. Heatmap created using Fieldtrip http://www.fieldtriptoolbox.org/ and Matlab 2019b (https://uk.mathworks.com).

The topographies of average regression coefficients for the saccade-related gradients and stimulus-related gradients are plotted in Fig. 3A. The extent to which the Saccade-only and Stimulus-only conditions could account for the gradients measured in the Saccade + stimulus condition varied considerably across different areas (Fig. 3B). Overall, the variance explained was higher in right than in left lateralized sensors (t(27) = − 5.91, *p* < 0.001).

**Figure 4:**
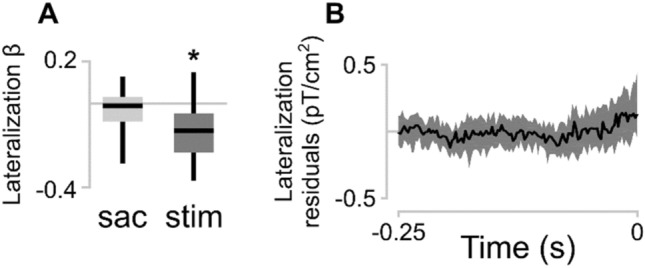
(**A**) Lateralization of the beta coefficients. The median beta coefficient of right lateralized sensors was subtracted from the median beta coefficient of the left lateralized sensors. Boxplots represent the median and dispersion across participants. Asterisk indicates that the mean significantly deviates from zero (*p* < 0.001). (**B**) Lateralization of residual planar gradients: residuals from sensors on the left side of the helmet minus the residuals from sensors on the right side. Line represents the median; shaded area represents the interquartile range across participants.

The beta coefficients for the Stimulus-only condition were stronger in right lateralized sensors (t(27) = − 4.78, *p* < 0.001; Fig. 4A). This is expected because we presented the stimulus in the left visual field, which means that it would be processed by the right visual cortex. The beta coefficients Saccade-only condition were not significantly lateralized (t(27) = − 1.58, *p* = 0.125).

Next, we examined the residual planar gradients (Fig. 4B). The residuals represent part of the planar gradient in the Saccade + stimulus condition that cannot be accounted for by a (scaled) combination of the Saccade-only and Stimulus-only gradients. From 112 ms before saccade onset, average residual planar gradients in multiple sensors were significantly above 0 (α = 0.05; Fig. 3C). The number of sensors with significant residual planar gradients increased over time to saccade onset to a maximum of 66 sensors. The topographic distribution of significantly deviating planar gradients was widespread. We tested potential lateralization of residuals by subtracting the median residuals in all right-lateralized sensors from all left-lateralized sensors. There was no significant difference (α = 0.05) between left and right lateralized sensors at any timepoint. We repeated the lateralization analysis for occipital and parietal sensors separately to be more sensitive to the areas where the largest residuals appear to be. There was no significant lateralization in either group of sensors. Moreover, we assessed potential differences between the number of significantly active sensors in the left and right hemisphere by means of chi-square tests and Bayes factor (BF). We used the Bayesian analysis of proportions implemented in the R package ‘BayesFactor’^52,53^. The number of right-lateralized significant sensors was not different than left-lateralized significant sensors between 112 and 50 ms before saccade onset (chi-square test, X(1) = 4.54, ns after Bonferroni correction; BF = 0.34, anecdotical evidence in favor of H0, not conclusive), and between 50 ms and saccade onset (chi-square test, X(1) = 3.87, ns after Bonferroni correction, BF = 0.57, anecdotical evidence in favor of H0, not conclusive).

Although unlikely, these results could be driven by noise (e.g., SQUID system noise). As a control analysis we recomputed the saccade-related beta coefficient for the trials belonging to the first and second half of the experimental sessions separately. We found similar results concerning the residual analysis and statistical comparison of the beta coefficients of the two groups revealed no significant differences, meaning that the noise level was constant across the experimental session and conditions.

In sum, we extracted planar gradients that were potentially related to presaccadic processing of visual information. We defined remapping related planar gradients by taking the residuals from the Saccade + stimulus condition after regressing out the stimulus-related and the saccade-related planar gradients. Following the results of the EEG studies of Parks and Corballis^11,21^, we expected the residuals to be ipsilateral to the stimulus shortly before saccade onset. In our data, the residual planar gradients increased in strength closer to saccade onset, but globally, there was no significant difference between residual planar gradients over the left and in the right hemispheres.

## Discussion

Neurophysiological studies have demonstrated changes in receptive field properties of neurons that are related to the anticipation of the shift in visual input induced by saccades^1,5^. EEG studies have attempted to find a correlate of this process in humans by comparing significant differences in activity between conditions^11,21–23^. Here, we investigated event-related responses shortly before saccade onset with MEG, specifically with planar gradiometers. Instead of comparing differences between experimental conditions, we modelled the planar gradients evoked by a visual stimulus before saccade onset with the gradients evoked by a visual stimulus in the absence of a saccade and the gradients evoked by a saccade in the absence of a visual stimulus. We allowed the gradients in the two conditions to be linearly scaled to account for absolute response differences between conditions. We then subtracted the scaled visually-evoked and saccade-evoked responses, and further analyzed the residuals from the condition in which the visual stimulus was followed by a saccade. These residuals are unique to this latter condition and could therefore reflect saccade-modulated visual responses. Following two EEG studies^11,21^, it could be expected that the residuals would be greater over the ipsilateral hemisphere, where remapping would take place in anticipation of the saccade-induced visual changes. Instead, we observed a bilateral spread of residual gradients before saccade onset, in the absence of a clear lateralization.

The residual gradients that we observe in both hemispheres are relatively widespread. We attribute this considerable spread of activity to a combination of factors: the resolution of the measurement (MEG signal), the source of the effect itself and the experimental design adopted. It is important to note that these are not mutually exclusive alternatives. The spatial resolution of MEG is generally lower compared to other techniques, such as functional MRI^54^. Although single neurons and neuronal ensembles like the pinwheel columnar structures in primary visual cortex at the millimeter and sub-millimeters scale are known to be sensitive to these visual features, the spatial scale of MEG does not allow to access sub-millimeters scales. We speculate that MEG sensitivity to orientation and spatial frequency relies on nonuniform distribution of spatial frequency-selective neurons^55^, or a global different spatial frequency or orientation tuning between different visual cortical areas^56^.

Regarding the experimental design: we do not have access to individual receptive fields, but rather to global activity observed in each experimental condition. Like other similar experiments did previously^26^, our experimental design was built to assess the presence of a pre-saccadic effect. In these designs a potential pre-saccadic effect can be inferred from a shift in activity between hemispheres, thus an inherently widespread signal. We therefore argue that the effect we report likely reflects a combination of factors, as the resolution of the measurement (MEG signal), the source of the effect itself and the experimental design adopted, tailored towards a shift in activity between hemispheres. It is important to note that this activity shift might be indicative of a predictive shift of a population of neurons, manifested in a widespread, rather than in localized activity.

The previous EEG studies on the mechanisms underlying remapping involved a wide range of stimulus sizes and durations which could partially explain the inconsistent findings in the literature. In particular, two of the studies used flashed stimuli that disappeared prior to saccade onset^22,23^. On the other hand, for Parks and Corballis^11,21^ and in our study, the stimulus remained visible on the screen across the saccade. The case of stable objects across eye movements most closely matches real-life experience of visual stability with objects that are themselves spatially stable across saccades. However, using a flashed probe stimulus might better reflect a momentary state of the remapping process. The measurability of a remapping-related signal has indeed been demonstrated (both with single cell recordings and fMRI) to be sensitive to subtle changes to the experimental paradigm^57,58^.

In our study, participants made saccades immediately after the stimulus had appeared. Even though we only analyzed trials in which the saccade latency was at least 150 ms, this was still substantially shorter than the pre-saccadic fixation durations in several of the previous studies, which was in the order of a full second^11,21^. Moreover, the visual stimulus in this study appeared and disappeared within one second. One possibility is that spatiotopic representations build over time, across multiple fixations, over a period of seconds^59,60^. If so, then our results may reflect the transient remapping process but not capture all aspects of spatiotopic perception for stable objects, which might include more ipsilateral activity. However, as we have previously argued^34^, given that visual processing and the oculomotor system are fast, then remapping should also be fast in order to contribute to perceptual stability across a single saccade.

Our findings can be considered in light of the different theories of how we maintain stable and continuous perception across saccades. At the most abstract level, our results are consistent with theories of active perception and sensorimotor integration: neural activity is different when there is both a saccade and a stimulus, showing an interaction of the visual and the motor components^28–33^. The current results support, on a whole-brain level, previous neurophysiological evidence that oculomotor planning and responses to visual stimuli interact. There is growing evidence that making a saccade alters visual processing around the time of saccades, including peri-saccadic reduction but also post-saccadic enhancement in the responses of visual neurons^31,61,62^. For instance, saccades also alter oscillatory activity, in particular in the theta band^63,64^. Here, we observed significant changes in the magnetic fields starting around 112 ms prior to saccade onset that are consistent with these visuo-motor interactions. These findings therefore fit well with active perception theories and reveal a close alignment between vision and the oculomotor systems^65^.

Additionally, the observation that the neural modulation was bilateral suggests that when there is an attended object, saccade preparation may modulate both the current neurons involved in object processing and the future neurons that will soon be processing that object and benefit from a “preview” of that object (for review:^66^. Why would neurons in contralateral areas (soon to be ipsilateral after the saccade) be modulated? One possibility is that they will continue to represent the object well into the start of the new fixation, in order to perform a “soft hand-off” for an attended object that changes its retinotopic location across the saccade^13^. In terms of visual features such as spatial frequency, the spatial updating may be pre-saccadic but the neurons supporting feature processing (as indexed by spatial frequency decoding in^13^, may remain the same, without spatial remapping. This hand-off (or double spotlight, in the case of attention), requires the same neurons to effectively do two things (i.e. respond to both pre-saccadic location for a stimulus no longer in its receptive field and process new information), perhaps via multiplexing^67–69^.

Similarly, it has been argued that the updating of spatial attention, whether to an object or an empty location, involves a two-stage process with the first being predictive and pre-saccadic and the second stage being slower and post-saccadic^14^. One potential reason for the strong saccade + stimulus contralateral activity found here may be the modulation of the feature-selective neurons in order to maintain the feature representations across the saccade.

Given that objects in the real world are relatively stable (they do not suddenly appear or disappear around saccade onset), maintaining the pre-saccadic visual feature information across the saccade might be efficient and it has been shown that such pre-saccadic information reduces post-saccadic responses^70,71^. Thus, it makes sense that both the current and future receptive fields involving in processing an attended object might be involved in this predictive process. Our current results suggest that visual areas that process the stimulus at its pre-saccadic location and the areas that process the stimulus at its post-saccadic location may both play a role in establishing perceptual stability, at least in the context where the stimulus is behaviorally relevant.

## Data Availability

All data conducted in the context of this study is freely available on https://osf.io/ngud8/.
